# Preparation, Characterization, and Properties of Novel Ti-Zr-Be-Co Bulk Metallic Glasses

**DOI:** 10.3390/ma13010223

**Published:** 2020-01-04

**Authors:** Pan Gong, Fangwei Li, Junsong Jin

**Affiliations:** 1 State Key Laboratory of Materials Processing and Die & Mould Technology, Huazhong University of Science and Technology, No. 1037 Luoyu Road, Wuhan 430074, China; pangong@hust.edu.cn (P.G.);; 2 State Key Laboratory of Advanced Design and Manufacturing for Vehicle Body, Hunan University, Changsha 410082, China

**Keywords:** bulk metallic glasses, titanium alloys, glass-forming ability, thermoplastic formability, compressive properties, crystallization behavior, corrosion resistance

## Abstract

We developed novel Ti-Zr-Be-Co bulk metallic glasses through Co addition based on a ternary Ti_45_Zr_20_Be_35_ alloy. By altering the alloying routes and alloying contents, the influence of Co alloying on glass-forming ability, thermal stability, thermoplastic formability, crystallization behavior, and corrosion resistance has been investigated systematically. It was found that the best alloying route for enhancing the glass-forming ability, thermoplastic formability, compressive plasticity, and corrosion resistance is to replace Be by Co. Ti_45_Zr_20_Be_23_Co_12_ possesses the largest critical diameter of 15 mm for glass formation. Ti_45_Zr_20_Be_27_Co_8_ possesses the highest thermoplastic formability which is comparable to that of Vitreloy alloys. Ti_45_Zr_20_Be_25_Co_10_ exhibits the largest room temperature plasticity of 15.7% together with a high specific strength of 3.90 × 10^5^ Nm/kg. The addition of Co also strongly affects the crystallization behavior of the base alloy, resulting in a more complex crystallization process. The corrosion resistance of Ti-Zr-Be alloy in 1 mol/L HCl solution can also be enhanced by Co alloying. The related mechanisms have been explained in detail, which provide guidance for the composition design of Ti-based metallic glasses with improved properties.

## 1. Introduction

Due to the demand of reducing the energy consumption and protecting the environment, lightweight metals and alloys are attached more and more importance in a broad range of industries such as aerospace, electronics, automotive, etc. [1,2]. Bulk metallic glasses (BMGs) possess better mechanical properties and corrosion resistance than those of the corresponding crystalline alloys due to the single amorphous structure [3,4,5]. Thus, light-metal-based BMGs have attracted tremendous attention and research. In the past decades, many lightweight BMGs such as Al- [6,7], Mg- [8,9], and Ti-based BMGs [10,11,12,13,14] have been successfully developed. However, the main bottleneck of Al-based BMGs is the relatively low glass-forming ability (GFA) (the maximum critical diameter of only 1.5 mm [6]), while Mg-based BMGs always lack room temperature plasticity even upon compression [8,9]. In contrast, Ti-based BMGs possess the most promise for engineering application because of their relatively good combination of GFA, strength, and ductility [10,11,12,13,14]. However, as the developed Ti-based BMGs are still far from perfect, to widen the application scopes, more efforts are needed to develop novel Ti-based BMGs with higher glass-forming ability (GFA), larger room temperature plasticity, and better corrosion resistance.

The alloying method is known as a widely used way to developed novel alloys with enhanced properties [15,16]. For BMGs, it has been found that the GFA, mechanical properties, and corrosion resistance can be improved by adding alloying elements with optimized contents [17,18,19,20]. Zhu et al. research shows that Sn alloying is effective to enhance the GFA and plasticity of Ti-Zr-Cu-Pd BMGs [21]. Gong et al. [22,23] and Zhao et al. [24] reported that Fe (Al or Ni) addition effectively improves the GFA and mechanical properties of Ti-Zr-Be BMGs. Thus, for the composition design of novel Ti-based BMGs, the appropriate base alloy and alloying element should be determined first. In this study, we chose a Ti-Zr-Be ternary BMG as the starting alloy because this alloy exhibits relatively low density, high specific strength, and good GFA compared with other Ti-based ternary alloys [10]. Regarding the selection of the alloying element, it is known that Co-based BMGs possess the highest strength among the developed BMGs [25], implying that Co is an effective element for developed alloys with high strength. More importantly, according to our previous study, Fe alloying is an effective alloying element for Ti-Zr-Be BMGs [23]. As Fe and Co possess similar properties [26], in this study, Co was selected as an addition to develop novel quaternary Ti-based metallic glasses. Different combinations of adding routes and alloying contents have been attempted for the optimization of alloy composition. The effects of Co alloying on the GFA, thermal stability, room temperature mechanical properties, thermoplastic formability, crystallization kinetics, and corrosion resistance have been systematically investigated and the related mechanisms have also been proposed.

## 2. Materials and Methods

The pre-alloyed ingots of Ti_45_Zr_20_Be_35−*x*_Co_*x*_ (*x* = 0, 2, 4, 6, 8, 10, 12, 14, and 16 at%), Ti_45_Zr_20−*x*_Be_35_Co_*x*_ (*x* = 2, 4, and 6 at%), and (Ti_45_Zr_20_Be_35_)_100−*x*_Co_*x*_ (*x* = 2, 3, 4, and 6 at%) were prepared by arc melting with the protection of high-purity argon atmosphere. Pure elements (>99.9 wt%) were used as the starting materials. Pure titanium ingot was melted before the melting of alloy ingots to absorb the residual oxygen. The mass of the master ingots was about 20 g. The ingots were remelted assisted by electromagnetc stirring six times to get compositional homogeneity. Then, as-cast conical samples and as-cast rods were fabricated by copper mold tilt-pour casting and copper mold injection casting, respectively. To evaluate the GFA of different Ti-Zr-Be-Co alloys more efficiently, sections with different diameters were cut from the conical samples using a diamond saw. Then, the sections were examined by X-ray diffraction (XRD, Rigaku D/max-RB, Tokyo, Japan) with Cu Kα radiation and transmission electron microscope (TEM, FEI Tecnai G20, Hillsboro, OR, USA). The TEM sample was prepared by twin-jet polishing. Thermal analysis were characterized (gas for protection: 99.995 wt% Ar) at different heating rates (5, 10, 20, and 40 K/min) using differential scanning calorimetry (DSC, Netzsch STA 409 C/CD, Selb, Germany). Room temperature mechanical properties were evaluated by compression tests using a Zwick-Z020 testing machine (Ulm, Germany) (strain rate: 4 × 10^−4^ s^−1^; sample size: *ϕ* 2 × 4 mm). The LEO-1530 scanning electron microscope (Oberkochen, Germany) was used to observe the lateral and fracture surfaces of the fracture samples. The density of the samples was measured by the Archimides method using an analytical electronic balance (Sartorius BSA124S-CW, Gottingen, Germany, accuracy: ±0.1 mg). The thermoplastic formability of BMGs was evaluated by thermomechanical analysis (TMA, TA Q400EM, Lindon, UT, USA, sample size: *ϕ* 3 × 1.5 mm, heating rate: 20 K/min). A load of 1.2 N was applied on the sample during the whole process of TMA tests. A conventional three-electrode setup (the reference electrode: Saturated calomel electrode (SCE); the auxiliary electrode: Pt counter electrode) was deployed to study the electrochemical characteristics. The sweep rate for the anodic polarization measurement is 0.5 mV·s^−1^ and the diameter of the samples used is 4 mm. The measurement should start after open circuit immersion for 1 h to make the open circuit potential stable. For each composition, the corrosion tests should be repeated three times. The electrolyte used was 1 M HCl solution. Before electrochemical testing, all the specimens were mechanically ground using an SiC paper and carefully polished to a mirror surface. Then, the samples were ultrasonically cleaned using acetone, and finally air-dried for at least 24 h.

## 3. Results and Discussion

### 3.1. GFA and Thermal Stability

**Figure 1:**
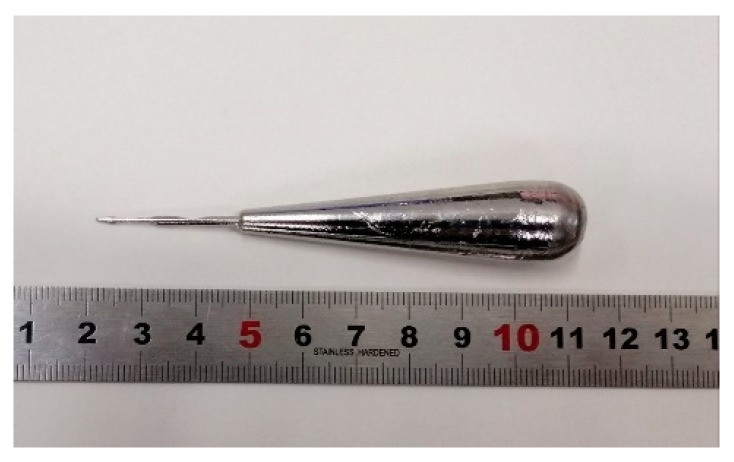
An as-cast conical sample for determining the critical diameter of different alloy compositions.

**Figure 2:**
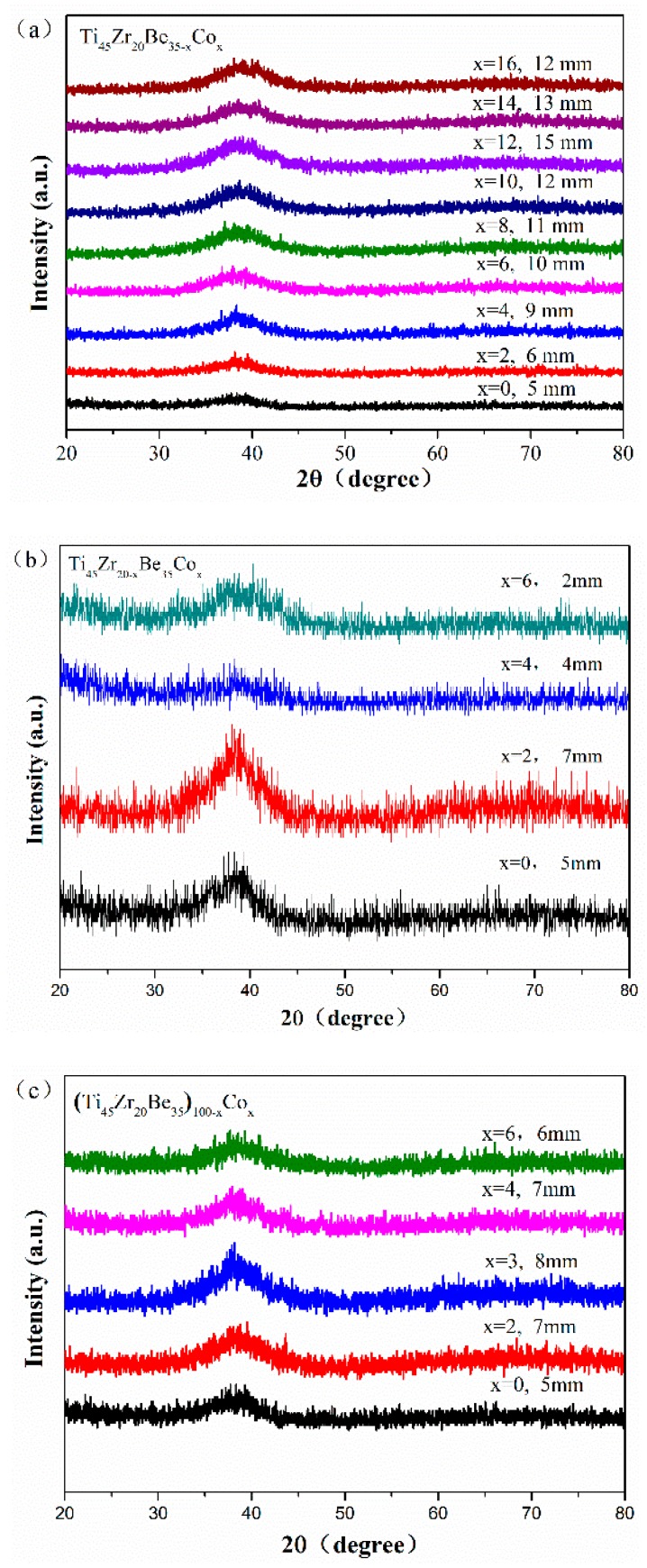
XRD patterns of as-cast. (**a**) Ti_45_Zr_20_Be_35−*x*_Co_*x*_ (*x* = 0, 2, 4, 6, 8, 10, 12, 14, and 16), (**b**) Ti_45_Zr_20−*x*_Be_35_Co_*x*_ (*x* = 0, 2, 4, and 6), and (**c**) (Ti_45_Zr_20_Be_35_)_100−*x*_Co_*x*_ (*x* = 0, 2, 3, 4, and 6) sections with their critical diameters.

**Figure 3:**
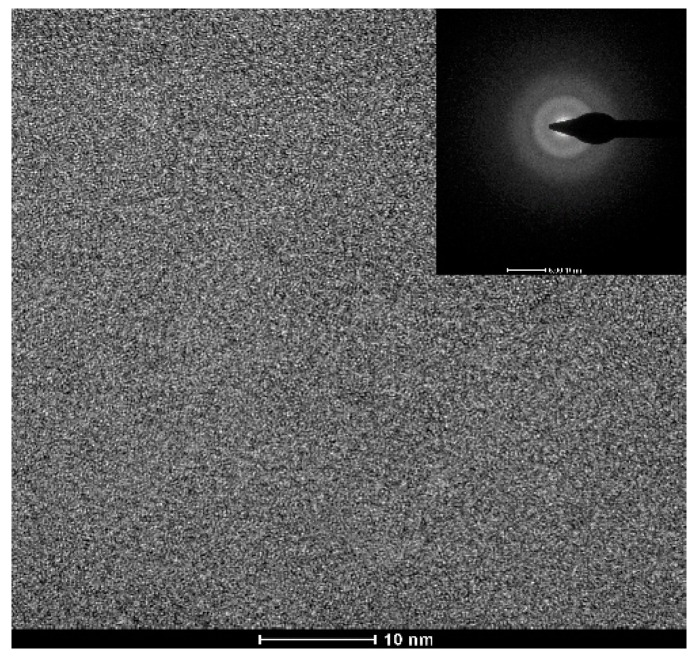
TEM bright field image with an SAED pattern for the 15 mm diameter Ti_45_Zr_20_Be_23_Co_12_ rod.

Figure 1 shows the appearance of an as-cast conical sample, which possesses a maximum diameter of 15 mm. XRD tests were conducted to preliminarily determine critical diameter *D*_c_ for glass formation base on a critical state under which the XRD pattern only consists of broad diffraction halos, as explained in Figure S1. Figure 2 displays the XRD spectra of the Ti-Zr-Be-Co sections with different diameters. The critical diameter of the Co-free alloy is 5 mm. It is found that the substitution Co for Be first significantly improve the GFA of Ti_45_Zr_20_Be_35_ BMG and then deteriorates the GFA with Co content increasing. The optimized Co content is determined to be 12 at%, and the critical diameter increases to >15 mm. To further confirm the fully amorphous structure of the 15 mm diameter Ti_45_Zr_20_Be_23_Co_12_ section, high-resolution TEM tests were conducted. Both of the HRTEM image and the corresponding selected electron diffraction pattern exhibit characteristics of fully amorphous structure (as shown in Figure 3). As the maximum diameter of our conical sample is 15 mm, the critical diameter of Ti_45_Zr_20_Be_23_Co_12_ alloy may be larger than 15 mm. However, the substitution of Co for Zr or Co doping directly first improves the GFA slightly and then degrades the GFA with the Co content increasing. The maximum critical diameters are 7 and 8 mm, respectively, which are much smaller compared with that of Ti_45_Zr_20_Be_23_Co_12_ BMG. Therefore, replacing Be by Co as the best alloying route is beneficial to develop Ti-Zr-Be-Co alloys with larger critical diameters for glass formation.

**Figure 4:**
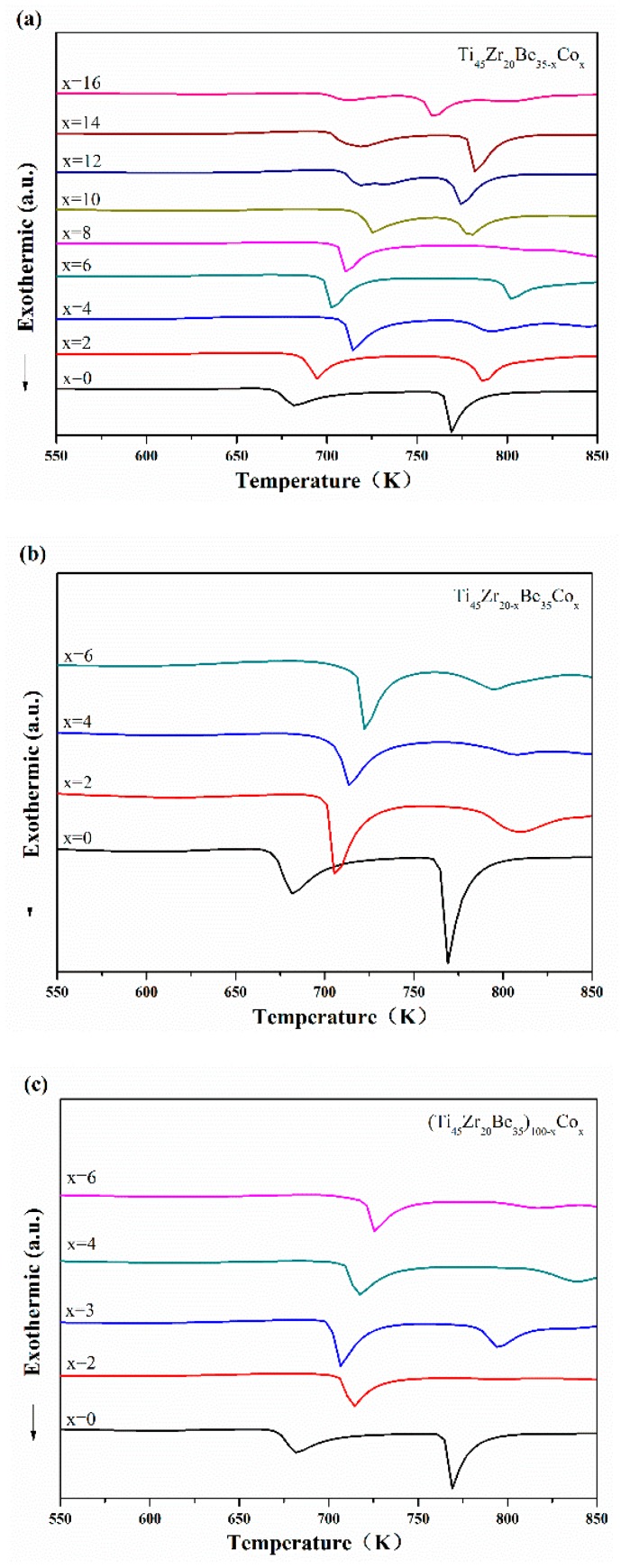
DSC curves of (**a**) Ti_45_Zr_20_Be_35−*x*_Co_*x*_ (*x* = 0, 2, 4, 6, 8, 10, 12, 14, and 16), (**b**) Ti_45_Zr_20−*x*_Be_35_Co_*x*_ (*x* = 0, 2, 4, and 6), and (**c**) (Ti_45_Zr_20_Be_35_)_100−*x*_Co_*x*_ (*x* = 0, 2, 3, 4, and 6) samples with different heating rates.

Continuous DSC tests were performed to further investigate the thermal properties of the developed Ti-based alloys. Figure 4 displays the DSC curves of Ti-Zr-Be-Co samples. The values of critical diameter *D*_c_, glass transition temperature *T*_g_, onset temperature for crystallization *T*_x_, width of supercooled liquid region Δ*T*_x_ (defined as Δ*T*_x_ = *T*_x_ − *T*_g_), and liquidus temperature *T*_l_ for Ti-Zr-Be-Co BMGs have been summarized in Table 1. With few exceptions, by partially replacing Be with Co, the values of *T*_g_, *T*_x_, and Δ*T*_x_ increase first and then decrease with increasing Co content. By partially replacing Zr with Co or directly Co doping, the values of *T*_g_, *T*_x_, and Δ*T*_x_ increase. The enlargement of supercooled liquid region indicates the enhancement of thermal stability induced by Co addition. Some of the Co containing alloys possess a significant supercooled liquid region (>100 K). By comparison, the supercooled liquid region of most developed Ti-based BMGs are much narrower [21,22,23,24]. No marked relationship between *D*_c_ and GFA indicators such as Δ*T*_x_, *T*_rg_ (defined as *T*_rg_ = *T*_g_/*T*_l_), and *γ* parameter (defined as *γ* = *T*_x_/(*T*_g_ + *T*_l_)) can be observed. Among all the studied alloys, the Ti_45_Zr_20_Be_23_Co_12_ alloy exhibits the highest GFA with a large critical diameter up to 15 mm.

We discuss the mechanisms of GFA improvement induced by Co additions in view of the change of mixing enthalpy Δ*H*_mix_ [27], atomic size difference *δ* [27,28], and electronegativity difference Δ*x* [28]. The methods of calculating Δ*H*_mix_, *δ*, and Δ*x* were proposed in References [27,28]. Table 2 lists the calculated values of Δ*H*_mix_, *δ*, and Δ*x* for the studied Ti-Zr-Be-Co BMGs. For all the three alloying routes, as the Co content is increased, the values of *δ* decreases and the value of Δ*x* increases. Zhao et al. [24] proposed an empirical rule for developing larger-sized Ti-based BMGs. They found that centimeter-sized Ti-based BMGs always possess a large value of *δ* (11.76 ≤ *δ* ≤ 13.33), relatively large value of Δ*x* (0.1194 ≤ Δ*x* ≤ 0.1837), and more negative enthalpies of mixing (–33.15 kJ/mol ≤ Δ*H*_mix_ ≤ −23.81 kJ/mol). As shown in Table 2, the values of related parameters for Ti-Zr-Be-Co BMGs with critical diameters up to a centimeter order all fall in the indicated range. Our experimental results further prove the effectiveness of the proposed rule. It was known that properly increasing the *δ* and Δ*x* is beneficial to enhance the GFA of BMGs [29,30,31,32,33]. Among the three alloying routes, the decreasing rate of *δ* is the slowest while the increasing rate of Δ*x* is the fastest for substitution of Be for Co. As a result, Ti_45_Zr_20_Be_35-x_Co_x_ alloys possess relatively better GFA. Conversely, the decreasing rate of *δ* is the fastest while the increasing rate of Δ*x* is the slowest for substitution of Zr for Co. Then, the Ti_45_Zr_20_Be_35−*x*_Co_*x*_ alloys possess a relatively poor GFA. According to Inoue’s three empirical rules [34], large negative heats of mixing also affects the glass formation of BMGs. As shown in Table 2, the developed centimeter-sized Ti-Zr-Be-Co alloys possess negative heats of mixing with large absolute values no less than 30.50 kJ/mol. The change of Δ*H*_mix_ induced by Co addition is relatively small so that the consequent influence is not as significant as those of *δ* and Δ*x*. Similar phenomena have been found in the Ti-Zr-Be-Fe alloy system [35].

### 3.2. Thermoplastic Formability

**Figure 5:**
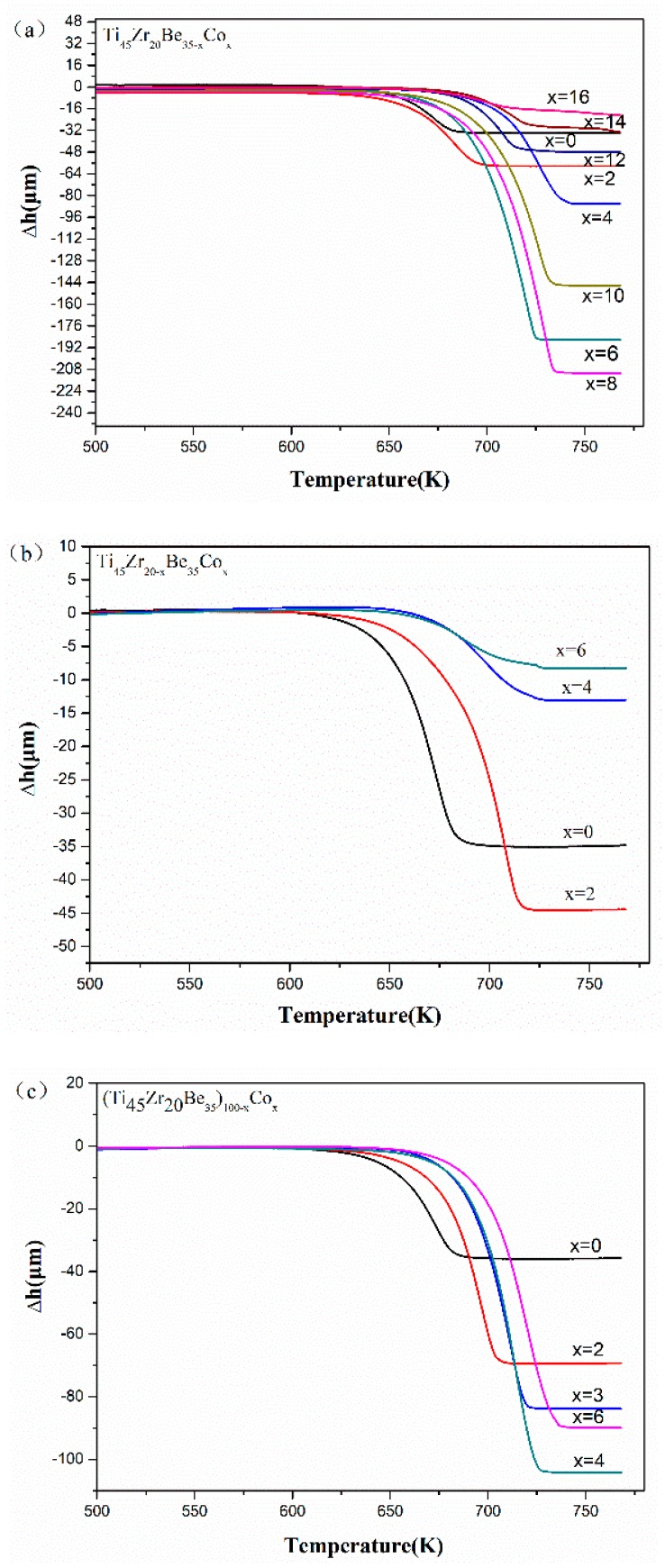
TMA results of (**a**) Ti_45_Zr_20_Be_35−*x*_Co_*x*_ (*x* = 0, 2, 4, 6, 8, 10, 12, 14, and 16), (**b**) Ti_45_Zr_20−*x*_Be_35_Co_*x*_ (*x* = 0, 2, 4, and 6), and (**c**) (Ti_45_Zr_20_Be_35_)_100−*x*_Co_*x*_ (*x* = 0, 2, 3, 4, and 6) samples.

The thermoplastic forming of Ti-based glassy alloys is more difficult compared with other developed BMGs (e.g., Pd- [36] and Zr-based BMGs [37,38]). Thus, it would be great to enhance the formability of Ti-based BMGs in their supercooled liquid state through the alloying method. Figure 5 shows the TMA results of the studied Ti-Zr-Be-Co glassy alloys. It can be found that for all the three alloying routes, the thermoplastic formability first enhances and then deteriorates with increasing Co content. By comparison, the Ti_45_Zr_20_Be_27_Co_8_ alloy exhibits the best thermoplastic formability indicated by the largest value of compressive deformation Δ*h* (210.74 mm) in the supercooled liquid region. The above results imply that substitution of Co for Be is the best choice to develop Ti-Zr-Be BMGs with better thermoplastic formability.

Schroers [39] proposed an *S* parameter (defined as *S* = Δ*T*_x_/*T*_l_ − *T*_g_) to evaluate the formability of BMGs in a supercooled liquid state. As shown in Table 3, although a larger *S* value does not necessarily mean better thermoplastic formability, the alloys which exhibit relatively better thermoplastic formability possess relatively large values of *S* compared with other parameters such as Δ*T*_x_, *T*_rg_, and *γ*. In this sense, the *S* parameter reflects the thermoplastic formability of studied Ti-based BMGs at least to a certain extent. For instance, the Ti_45_Zr_20_Be_27_Co_8_ alloy possesses an *S* value of 0.194, which is much larger compared with the base alloy (0.128) and other Ti-based BMGs (<0.14) [40,41,42], indicating that Co alloying is helpful to develop Ti-based BMGs with better thermoplastic formability.

### 3.3. Mechanical Properties

**Figure 6:**
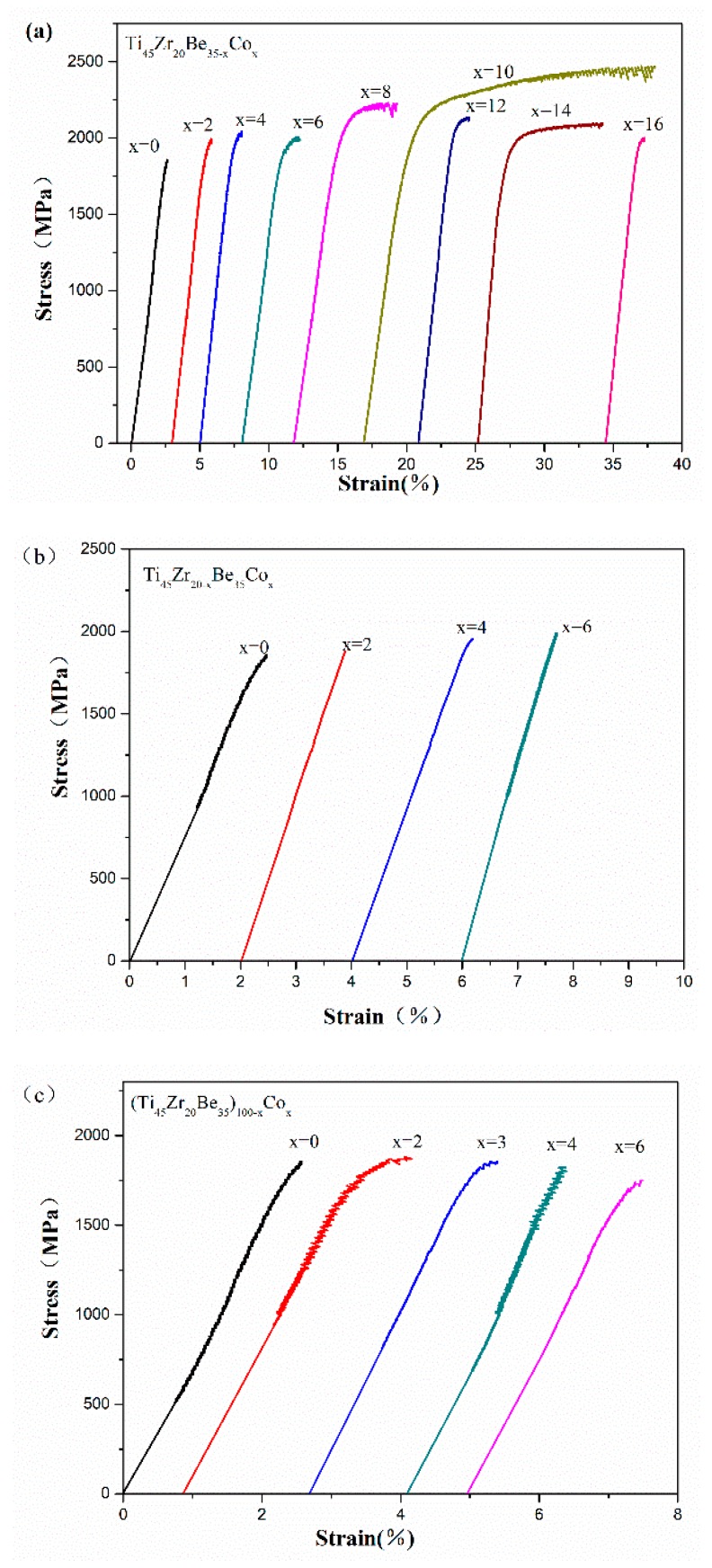
Compressive stress-strain curves of (**a**) Ti_45_Zr_20_Be_35−*x*_Co_*x*_ (*x* = 0, 2, 4, 6, 8, 10, 12, 14, and 16), (**b**) Ti_45_Zr_20−*x*_Be_35_Co_*x*_ (*x* = 0, 2, 4, and 6), and (**c**) (Ti_45_Zr_20_Be_35_)_100−*x*_Co_*x*_ (*x* = 0, 2, 3, 4, and 6) samples.

**Figure 7:**
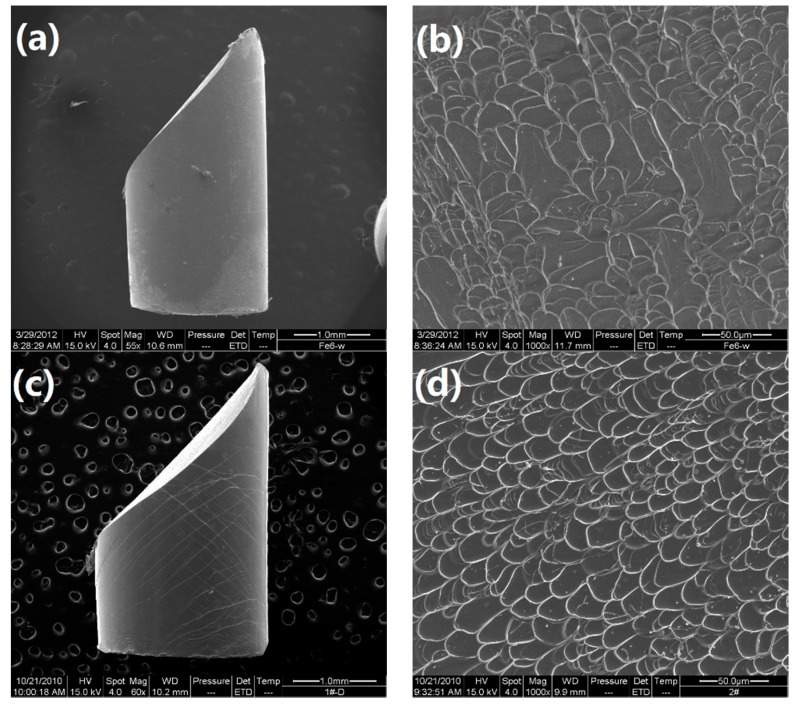
SEM images of fractured samples: (**a**) The lateral surface for the Ti_45_Zr_20_Be_35_ alloy; (**b**) the fractured surface for the Ti_45_Zr_20_Be_35_ alloy; (**c**) the lateral surface for the Ti_45_Zr_20_Be_25_Co_10_ alloy; (**d**) the fractured surface for the Ti_45_Zr_20_Be_25_Co_10_ alloy.

Room temperature compression tests have been conducted to evaluate the mechanical properties of the developed alloys. The typical compressive stress-strain curves are shown in Figure 6. Densities (*ρ*) and mechanical properties of the developed Ti-Zr-Be-Co BMGs, including the yield strength *σ*_y_, ultimate strength *σ*_max_, plastic strain *ε*_p_, and specific strength *σ*_sp_ (defined as *σ*_y_/*ρ*), are summarized in Table 4. According to Figure 6, for the base alloy, the compressive strength is much higher (1835 MPa) than that of crystalline Ti alloys but no obvious yielding can be observed. Substitution of Co for Zr or Co doping slightly enhanced the strength but the plasticity does not improve a lot. However, the addition of Co to replace Be enhances the compressive strength and improves the room temperature plasticity simultaneously. A prime example is that the yield strength and plastic strain of the Ti_45_Zr_20_Be_25_Co_10_ alloy are 2054 MPa and 15.7%, which are significantly enhanced compared with those of the base alloy. The SEM images of the fractured Ti_45_Zr_20_Be_35_ and Ti_45_Zr_20_Be_25_Co_10_ samples are shown in Figure 7. Both of the two samples exhibit a typical shear fracture mode. However, for the Ti_45_Zr_20_Be_25_Co_10_ sample, more jagged shear bands appear on the lateral surface, while the fractured surface exhibits well-developed vein patterns, implying a much better compressive plasticity. Although the density is slightly higher, the Ti_45_Zr_20_Be_25_Co_10_ alloy possesses a high specific strength of 3.90 × 10^5^ Nm/kg, which is enhanced by 0.8% compared with that of the base alloy.

Our experimental results show that no matter which alloying route is chosen, the compressive strength is enhanced by Co alloying. It has been found that with the increase of *δ*, the topological ordering can be enhanced, resulting in the formation of highly packed structure and the enhancement of strength [28]. However, according to Table 2, the value of *δ* decreases as the Co content increases, so that the compressive strength of Ti-Zr-Be-Co alloys may be affected by other factors. Yang et al. [43] found that the BMGs which possess higher value of *T*_g_ always exhibit higher strength. Thus, the enhancement of strength induced by Co alloying could be mainly related to the increase of *T*_g_.

The improvement of room temperature compressive plasticity can be explained from two aspects. First, it is already known that increasing the Poisson’s ratio is beneficial to release the shear localization during the deformation process of BMGs [44]. The Poisson’s ratio of Co (0.31) is much larger than that of Be (0.032), but slightly smaller than those of Zr (0.34) and Ti (0.32). In this sense, replacing Be by Co should be the best alloying route to increase the Poisson’s ratio and improve the room temperature plasticity. Second, Zr possesses the largest atomic size compared with other constituent elements, so the free volume may concentrate around the Zr–Zr atomic pairs [45,46,47]. Thus, the free volume content is strongly affected by the Zr content. Replacing Be by Co does not decrease the Zr content while the other two alloying routes both decrease the Zr content to varying degrees. To sum up, substitution of Be by Co may increase the value of Poisson’s ratio and maintain the free volume content, which contributes to an improvement of room temperature compressive plasticity.

### 3.4. Activation Energies

**Figure 8:**
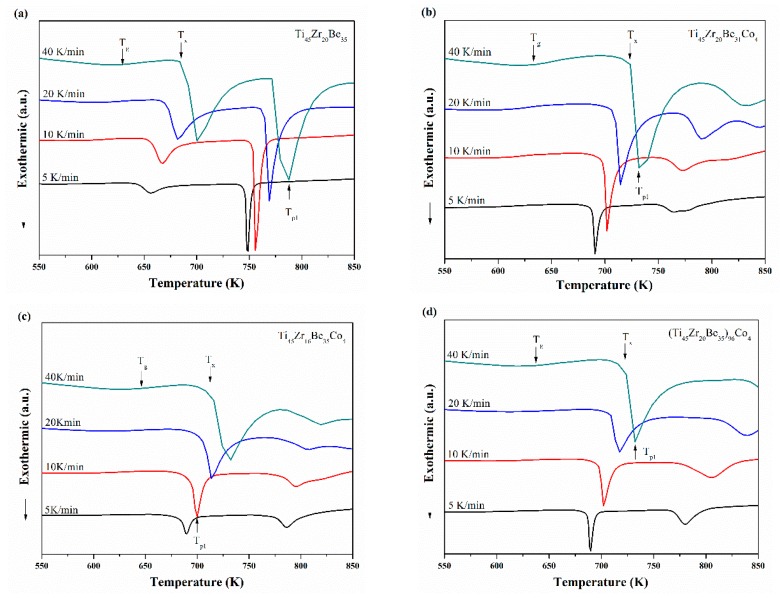
Continuous DSC curves for (**a**) Ti_45_Zr_20_Be_35_, (**b**) Ti_45_Zr_20_Be_31_Co_4_, (**c**) Ti_45_Zr_16_Be_35_Co_4_, and (**d**) (Ti_45_Zr_20_Be_35_)_96_Co_4_ BMGs.

Ti_45_Zr_20_Be_35_, Ti_45_Zr_20_Be_31_Co_4_, Ti_45_Zr_16_Be_35_Co_4_, and (Ti_45_Zr_20_Be_35_)_96_Co_4_ BMGs were chosen for non-isothermal crystallization analysis. The continuous DSC plots for these four alloys with different heating rates are shown in Figure 8a–d, which exhibit a clear endothermic glass transition and a complex multi-step crystallization process. With increasing heating rate, *T*_g_, *T*_x_, and *T*_p1_ (the peak temperature of the first crystallization event) shift to higher temperatures and the supercooled liquid region becomes broader. The activation energies at a specific heating rate can be calculated using the commonly used Kissinger and Moynihan equations, respectively. The former is expressed as [48]:

(1) $$\ln(\frac{\beta }{T^{2}})=-\frac{E}{RT}+C_{1}$$

The Moynihan equation is given as [49]:

(2) $$\ln(\beta )=-\frac{E}{RT}+C_{2}$$

where, *β* is the heating rate adopted, *T* is the related characteristic temperatures such as *T*_g_, *T*_x_, and *T*_p1_, *E* represents the corresponding activation energy, *R* is the ideal gas constant, *C*_1_ and *C*_2_ are two constants.

**Figure 9:**
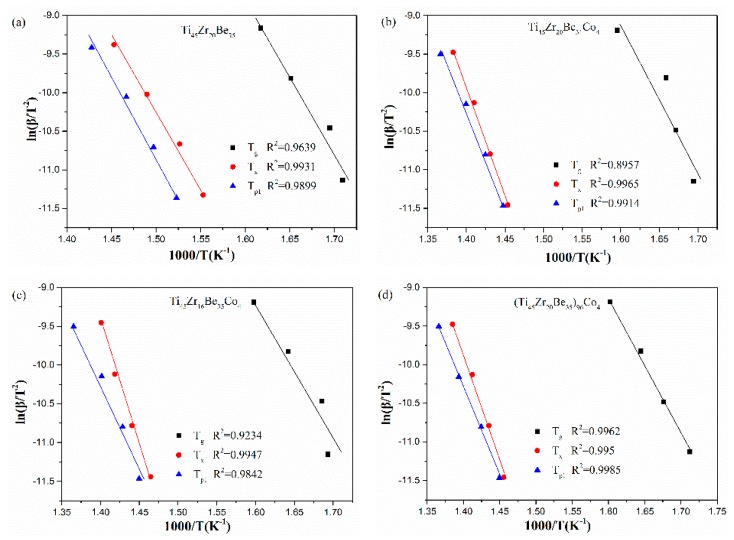
Kissinger plots for (**a**) Ti_45_Zr_20_Be_35_, (**b**) Ti_45_Zr_20_Be_31_Co_4_, (**c**) Ti_45_Zr_16_Be_35_Co_4_, and (**d**) (Ti_45_Zr_20_Be_35_)_96_Co_4_ BMGs.

**Figure 10:**
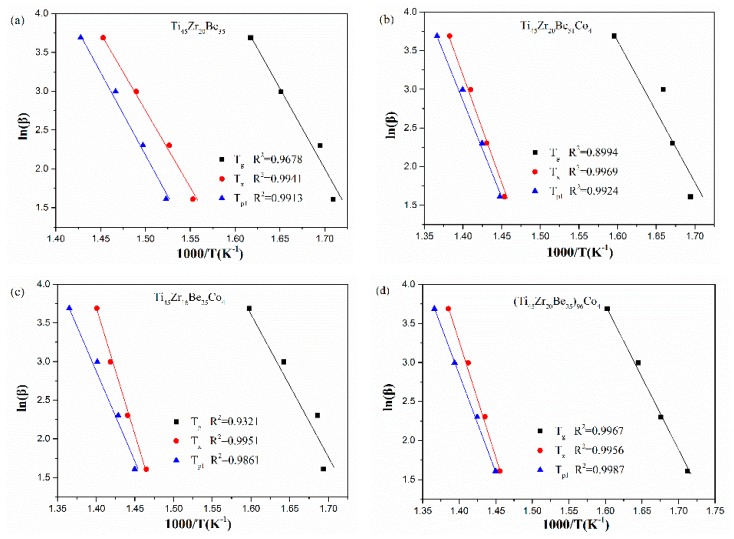
Moynihan (Ozawa) plots for (**a**) Ti_45_Zr_20_Be_35_, (**b**) Ti_45_Zr_20_Be_31_Co_4_, (**c**) Ti_45_Zr_16_Be_35_Co_4_, and (**d**) (Ti_45_Zr_20_Be_35_)_96_Co_4_ BMGs.

The corresponding Kissinger and Moynihan plots are shown in Figure 9 and Figure 10, respectively. By linear fitting, the values of *E*_g_, *E*_x_, and *E*_p1_ (activation energies corresponding to *T*_g_, *T*_x_ and *T*_p1_, respectively) were calculated and given in Table 5 and Table 6. For *E*_g_ and *E*_x_, it is observed that the values obtained by Kissinger and Moynihan equations are similar. With the addition of Co, the value of *E*_g_ decreases while the value of *E*_x_ increases. Among the four studied alloys, the Ti_45_Zr_16_Be_35_Co_4_ alloy possesses the largest value of *E*_x_ while the (Ti_45_Zr_20_Be_35_)_96_Co_4_ alloy possesses the lowest value of *E*_g_. Thus, the (Ti_45_Zr_20_Be_35_)_96_Co_4_ alloy may possesses less stable glass structure, while the Ti_45_Zr_16_Be_35_Co_4_ alloy may has a higher thermodynamic stability.

Ozawa and Boswell equations are also widely used for evaluating the activation energies of crystallization for BMGs. The Ozawa equation can be written as [50]:

(3) $$\ln(\beta )=-1.0516\frac{E}{RT_{p}}+C_{3}$$

The Boswell equation is defined as [51]:

(4) $$\ln(\frac{\beta }{T_{p}})=-\frac{E}{RT_{p}}+C_{4}$$

where, *T*_p_ is the peak crystallization temperature, *E* is the activation energy of crystallization, *C*_3_ and *C*_4_ are constants.

**Figure 11:**
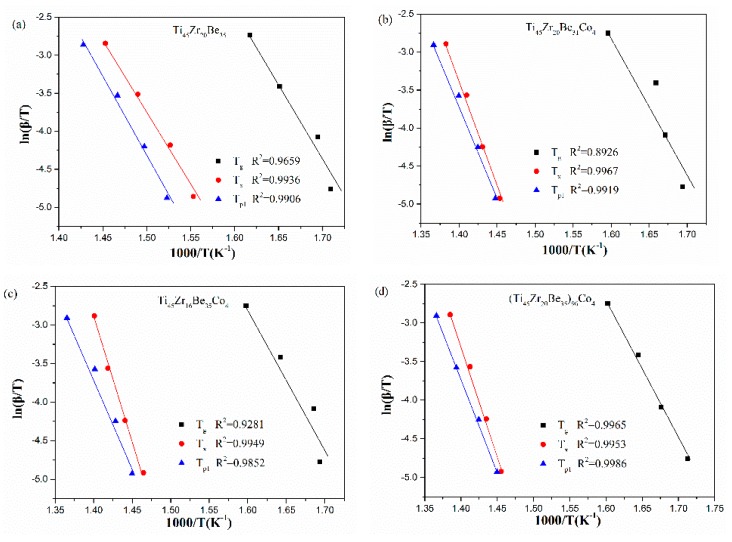
Boswell plots for (**a**) Ti_45_Zr_20_Be_35_, (**b**) Ti_45_Zr_20_Be_31_Co_4_, (**c**) Ti_45_Zr_16_Be_35_Co_4_, and (**d**) (Ti_45_Zr_20_Be_35_)_96_Co_4_ BMGs.

The Ozawa and Boswell plots for the studied four alloys are displayed in Figure 10 and Figure 11, respectively. The values of *E*_p1_ derived are also listed in Table 6. The values calculated by different equations are found to be similar. The Ti_45_Zr_20_Be_31_Co_4_ alloy possesses the highest *E*_p1_ value while the base alloy possesses the lowest value of *E*_p1_. As known, *E_x_* denotes the effective activation energy for nucleation, while *E*_p1_ is for grain growth [52]. In this sense, for the three Co-containing alloys, the energy barrier for the grain growth process is lower than that of the nucleation process, while the base alloy is diametrically opposed.

### 3.5. Non-Isothermal Crystallization Kinetics

The crystallized volume fraction *x* as a function of temperature can be determined as [53]:

(5) $$x=\frac{\int_{t_{0}}^{t}(dH_{c}/dt)dt}{\int_{t}^{t_{\infty }}(dH_{c}/dt)/dt}=\frac{A_{0}}{A_{\infty }}$$

where, *t_0_* and $t_{\infty }$ are the starting and ending times of crystallization, *dH_c_/dt* denotes the heat flow, and *A*_0_ and $A_{\infty }$ are the areas under DSC curves, respectively.

**Figure 12:**
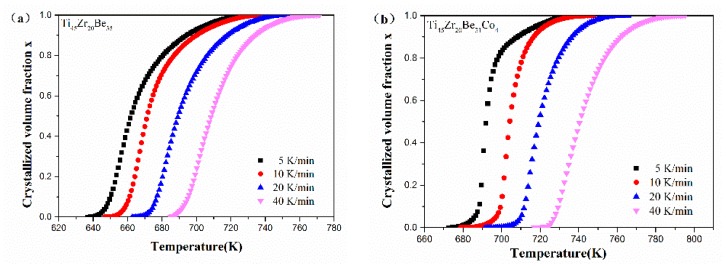

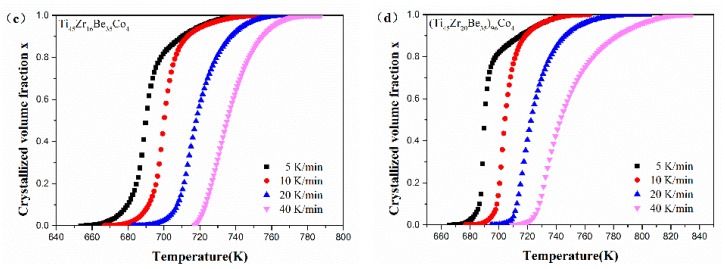
The crystallization volume fraction *x* as a function of temperature for (**a**) Ti_45_Zr_20_Be_35_, (**b**) Ti_45_Zr_20_Be_31_Co_4_, (**c**) Ti_45_Zr_16_Be_35_Co_4_, and (**d**) (Ti_45_Zr_20_Be_35_)_96_Co_4_ BMGs.

Figure 12 shows the values of *x* for the first crystallization event as a function of temperature for Ti_45_Zr_20_Be_35_, Ti_45_Zr_20_Be_31_Co_4_, Ti_45_Zr_16_Be_35_Co_4_, and (Ti_45_Zr_20_Be_35_)_96_Co_4_ BMGs. All the plots exhibit a typical sigmoid dependence on temperature, which is similar to that of other BMGs [54].

The isothermal crystallization kinetics are always analysed by the classic Johnson–Mehl–Avrami (JMA) equation [55]:

(6) $$\ln[-(1-x)]=n\ln(t-\tau )+C_{5}$$

Here, *t* is the time corresponding to the crystallized volume fraction, *τ* is the incubation time, and *C*_5_ is a constant.

Based on Equation (6), Nakamura et al. proposed an improved JMA equation for the non-isothermal process [56]:

(7) $$n(x)=\frac{Ad\ln[-\ln(1-x)]}{d\{\ln[(T-T_{0})/\beta ]\}}\;\mathrm{with}$$

(8) $$A=\frac{1}{1+E_{a}/RT(1-T_{0}/T)}$$

Here, *n*(*x*) represents the local Avrami exponent, *T*_0_ is the onset crystallization temperature, and *E_a_* is the activation energy.

**Figure 13:**
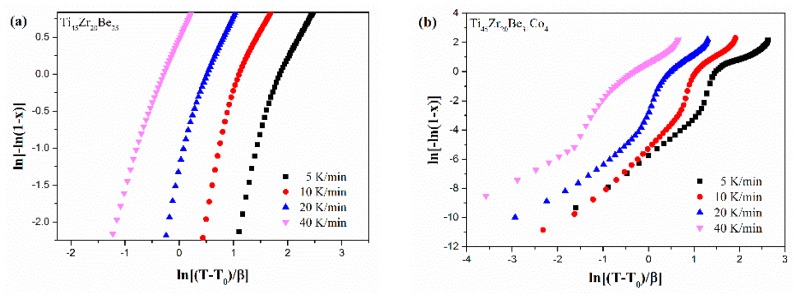

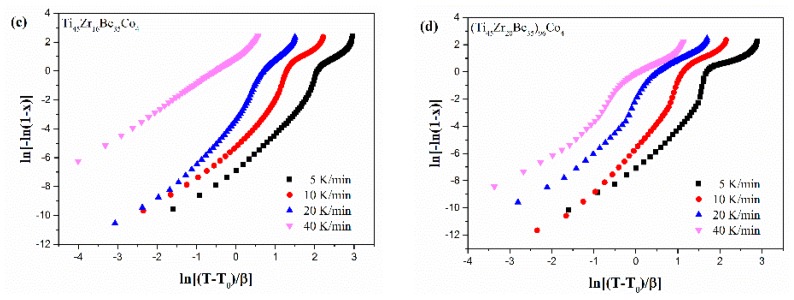
JMA plots for (**a**) Ti_45_Zr_20_Be_35_, (**b**) Ti_45_Zr_20_Be_31_Co_4_, (**c**) Ti_45_Zr_16_Be_35_Co_4_, and (**d**) (Ti_45_Zr_20_Be_35_)_96_Co_4_ BMGs (0.1 ≤ *x* ≤ 0.9).

**Figure 14:**
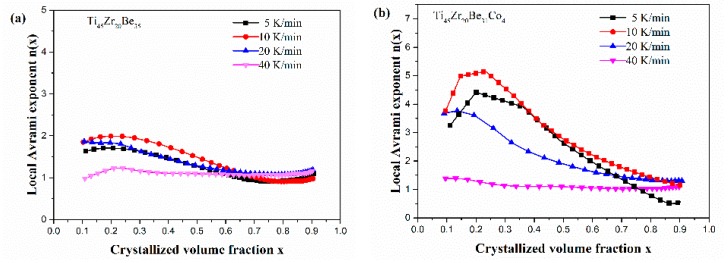

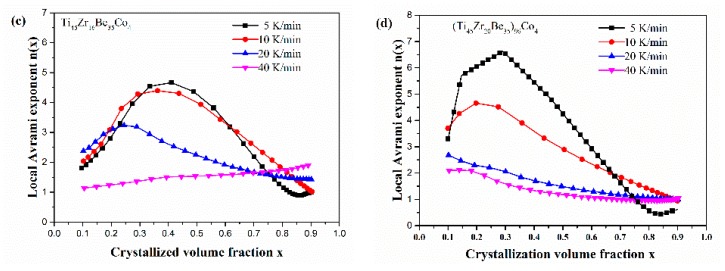
*n*(*x*) as a function of *x* for (**a**) Ti_45_Zr_20_Be_35_, (**b**) Ti_45_Zr_20_Be_31_Co_4_, (**c**) Ti_45_Zr_16_Be_35_Co_4_, and (**d**) (Ti_45_Zr_20_Be_35_)_96_Co_4_ BMGs (0.1 ≤ *x* ≤ 0.9).

Figure 13 shows the JMA plots for the studied four alloys (0.1 ≤ *x* ≤ 0.9). Table 7 lists the calculated values of *n*. The calculated values of *n* for the Co-free alloy are close to or smaller than 2.5. Thus, the first crystallization event of the base alloy is mainly dominated by a growth of primary crystal type with decreasing/constant nucleation rate [52,53,54]. However, the values of *n* for the three Co-containing are relatively larger, implying that the crystallization mechanism is the growth of small particles and the nucleation rate increases with time [52,53,54]. Compared with the base alloy, the Co-containing alloys possess higher values of *n*, implying that the addition of Co increases the nucleation rate. Among the three Co-containing alloys, Ti_45_Zr_20_Be_31_Co_4_ alloy possesses the largest average value of the Avrami exponent, indicating that this alloy possesses the highest nucleation rate. As the JMA plots shown in Figure 13 do not exhibit a single slope, the *n*(*x*) of the studied four alloys has also been studied to further understand the crystallization mechanisms (as shown in Figure 14). For the base alloy, the *n*(*x*) changes little during the crystallization process. However, with the addition of Co, the values of *n*(*x*) and variance amplitude of *n*(*x*) during the crystallization process both increase obviously. The *n*(*x*) of Co-containing alloys also exhibit a stronger dependence on the heating rate compared with the Co-free alloy. Thus, the crystallization becomes more complex with the addition of Co, which contributes to the improvement of GFA.

### 3.6. Corrosion Resistance

**Figure 15:**
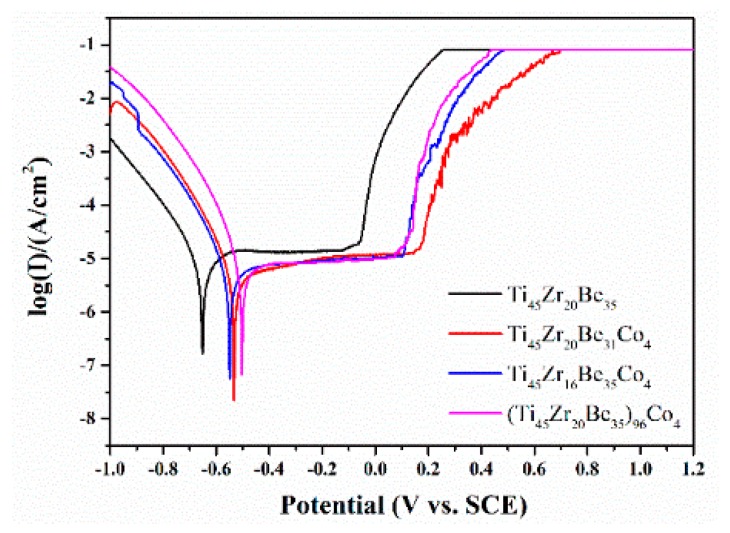
Potentiodynamic polarization curves of the Ti_45_Zr_20_Be_35_, Ti_45_Zr_20_Be_31_Co_4_, Ti_45_Zr_16_Be_35_Co_4_, and (Ti_45_Zr_20_Be_35_)_96_Co_4_ BMGs.

The Co alloying effect on the corrosion resistance was also studied by a potentiodynamic polarization test. Figure 15 shows the related potentiodynamic polarization curves. Table 8 lists the important electrochemical parameters such as corrosion potential (*E*_corr_), pitting potential (*E*_pit_), passive region width (*E*_pit_-*E*_corr_), corrosion current density (*i*_corr_), and polarization resistance (*R*_p_). It is found that the corrosion current densities for all the studied alloys are much lower than those of SUS321 stainless steel (1.377 × 10^−5^ A) and Fe_41_Co_7_Cr_15_Mo_14_C_15_B_6_Y_2_ amorphous alloy (2.46 × 10^−6^ A) [57], indicating that the studied alloys possess a high corrosion resistance in 1 mol/L HCl solution. Co-containing BMGs exhibit higher *E*_corr_ and *E*_pit_ together with lower *i*_corr_ than those of the base alloy, implying a better corrosion resistance because of the Co addition. The above experimental results are expectable as Co alloys possess high corrosion resistance [58,59]. However, the alloying route also strongly influences the corrosion resistance. It is known that chemical inert surface layers which possess a dense structure are easily formed on the surface of Ti and Zr [42]. In this sense, by substitution of Zr by Co or directly Co doping, although the addition of Co favors the enhancements of corrosion resistance, the content of Ti and/or Zr is diminished simultaneously, then the positive effect on the corrosion resistance induced by Co addition is weakened and not as strong as that for substitution of Be by Co. As a result, the Ti_45_Zr_20_Be_31_Co_4_ alloy possesses the lowest value of *i*_corr_ together with a largest value of passive region width, implying that this alloy exhibits the best corrosion resistance among the three studied Co-bearing alloys.

## 4. Conclusions

(1) The GFA of Ti_45_Zr_20_Be_35_ alloy is effectively improved by Co addition and the best alloying route is to replace Be by Co. Ti_45_Zr_20_Be_23_Co_12_ BMG possesses the best GFA among the developed Ti-Zr-Be-Co alloys with a critical diameter larger than 15 mm. The GFA improvement induced by Co alloying may result from a combination effect of many factors, e.g., atomic size, electrogenativity, heats of mixing, etc.
(2) Among the three alloying routes, substitution of Be by Co works best for enhancing the thermoplastic formability. Ti_45_Zr_20_Be_27_Co_8_ alloy possesses a large *S* value of 0.194, implying a much better thermoplastic formability compared with developed Ti-based BMGs.
(3) Replacing Be by Co is beneficial to improving the compressive strength and the plasticity simultaneously. Replacing Zr to Co and Co doping slightly enhances the strength but the plasticity does not improve obviously. The change of plasticity induced by Co addition can be explained in views of Poisson’s ratio and free volume content.
(4) Co addition increases the activation energies of crystallization but slightly decreases the activation energy of glass transition. Compared with the base alloy, the local Avrami exponent of the Co-containing alloys possesses larger values and exhibits stronger dependences on heating rate and crystallization volume fraction.
(5) Ti-Zr-Be-Co alloys possess better corrosion resistance compared with the Co-free alloy. The substitution of Co for Be achieves the best results on the improvement of corrosion properties.

## Figures and Tables

**Table 1:** Thermal parameters of Ti-Zr-Be-Co alloys (heating rate: 20 K/min, accuracy: ± 1 K).

Composition	*D*_c_ (mm)	*T*_g_ (K)	*T*_x_ (K)	*T*_l_ (K)	Δ*T*_x_ (K)	*T*_rg_	*γ*
Ti_45_Zr_20_Be_35_	5	589	671	1230	82	0.479	0.369
Ti_45_Zr_20_Be_33_Co_2_	6	599	684	1214	85	0.493	0.377
Ti_45_Zr_20_Be_31_Co_4_	9	603	709	1200	106	0.503	0.393
Ti_45_Zr_20_Be_29_Co_6_	10	598	698	1180	100	0.507	0.393
Ti_45_Zr_20_Be_27_Co_8_	11	596	706	1164	110	0.512	0.401
Ti_45_Zr_20_Be_25_Co_10_	12	611	718	1221	107	0.500	0.392
Ti_45_Zr_20_Be_23_Co_12_	15	642	709	1158	67	0.554	0.394
Ti_45_Zr_20_Be_21_Co_14_	13	634	701	1244	67	0.510	0.373
Ti_45_Zr_20_Be_19_Co_16_	12	640	698	1240	58	0.516	0.371
Ti_45_Zr_18_Be_35_Co_2_	7	592	701	1244	109	0.476	0.382
Ti_45_Zr_16_Be_35_Co_4_	4	589	705	1256	116	0.469	0.382
Ti_45_Zr_14_Be_35_Co_6_	2	601	717	1224	116	0.491	0.393
(Ti_45_Zr_20_Be_35_)_98_Co_2_	7	604	705	1246	101	0.485	0.381
(Ti_45_Zr_20_Be_35_)_97_Co_3_	8	602	699	1221	97	0.493	0.383
(Ti_45_Zr_20_Be_35_)_96_Co_4_	7	599	708	1188	109	0.504	0.396
(Ti_45_Zr_20_Be_35_)_94_Co_6_	6	605	719	1166	114	0.519	0.406

**Table 2:** The variation of parameters including Δ*H*_mix_, *δ*, and Δ*x* induced by Co alloying together with *D*_c_.

Composition	*D*_c_ (mm)	*δ*	Δ*H*_mix_ (kJ/mol)	Δ*x*
Ti_45_Zr_20_Be_35_	5	13.65	−30.94	0.0902
Ti_45_Zr_20_Be_33_Co_2_	6	13.45	−30.94	0.1039
Ti_45_Zr_20_Be_31_Co_4_	9	13.24	−30.93	0.1156
Ti_45_Zr_20_Be_29_Co_6_	10	13.03	−30.91	0.1259
Ti_45_Zr_20_Be_27_Co_8_	11	12.82	−30.87	0.1352
Ti_45_Zr_20_Be_25_Co_10_	12	12.60	−30.82	0.1435
Ti_45_Zr_20_Be_23_Co_12_	15	12.38	−30.76	0.1512
Ti_45_Zr_20_Be_21_Co_14_	13	12.15	−30.68	0.1583
Ti_45_Zr_20_Be_19_Co_16_	12	11.91	−30.59	0.1648
Ti_45_Zr_18_Be_35_Co_2_	7	13.56	−31.45	0.1007
Ti_45_Zr_16_Be_35_Co_4_	4	13.45	−31.82	0.1091
Ti_45_Zr_14_Be_35_Co_6_	2	13.32	−32.07	0.1159
(Ti_45_Zr_20_Be_35_)_98_Co_2_	7	13.60	−31.46	0.1034
(Ti_45_Zr_20_Be_35_)_97_Co_3_	8	13.57	−31.70	0.1092
(Ti_45_Zr_20_Be_35_)_96_Co_4_	7	13.54	−31.92	0.1145
(Ti_45_Zr_20_Be_35_)_94_Co_6_	6	13.48	−32.35	0.1243

**Table 3:** Experimental determined values of Δ*h* and *S* parameter of developed Ti-Zr-Be-Co BMGs.

Composition	Δ*h* (μm)	*S*
Ti_45_Zr_20_Be_35_	35.04	0.128
Ti_45_Zr_20_Be_33_Co_2_	58.09	0.138
Ti_45_Zr_20_Be_31_Co_4_	86.48	0.176
Ti_45_Zr_20_Be_29_Co_6_	185.72	0.172
Ti_45_Zr_20_Be_27_Co_8_	210.74	0.194
Ti_45_Zr_20_Be_25_Co_10_	146.55	0.175
Ti_45_Zr_20_Be_23_Co_12_	47.31	0.130
Ti_45_Zr_20_Be_21_Co_14_	29.78	0.110
Ti_45_Zr_20_Be_19_Co_16_	16.22	0.097
Ti_45_Zr_18_Be_35_Co_2_	44.46	0.167
Ti_45_Zr_16_Be_35_Co_4_	13.08	0.174
Ti_45_Zr_14_Be_35_Co_6_	8.76	0.186
(Ti_45_Zr_20_Be_35_)_98_Co_2_	69.26	0.157
(Ti_45_Zr_20_Be_35_)_97_Co_3_	83.61	0.157
(Ti_45_Zr_20_Be_35_)_96_Co_4_	104.09	0.185
(Ti_45_Zr_20_Be_35_)_94_Co_6_	89.74	0.203

**Table 4:** Measured densities and room temperature mechanical properties of developed Ti-Zr-Be-Co BMGs.

Composition	*σ*_y_ (MPa)	*σ*_max_ (MPa)	*ε*_p_ (%)	*ρ* (g/cm^3^)	*σ*_sp_ (N·m/kg)
Ti_45_Zr_20_Be_35_	1835 ± 44	1835	0	4.74 ± 0.01	3.87×10^5^
Ti_45_Zr_20_Be_33_Co_2_	1982 ± 35	1982	0.7 ± 0.1	4.96 ± 0.01	4.00×10^5^
Ti_45_Zr_20_Be_31_Co_4_	1882 ± 68	2021	1.0 ± 0.1	5.15 ± 0.01	3.65×10^5^
Ti_45_Zr_20_Be_29_Co_6_	1868 ± 57	2007	2.0 ± 0.1	5.17 ± 0.01	3.61×10^5^
Ti_45_Zr_20_Be_27_Co_8_	2074 ± 82	2201	5.6 ± 0.1	5.21 ± 0.01	3.98×10^5^
Ti_45_Zr_20_Be_25_Co_10_	2054 ± 45	2465	15.7 ± 0.1	5.26 ± 0.01	3.90×10^5^
Ti_45_Zr_20_Be_23_Co_12_	2056 ± 77	2136	1.5 ± 0.1	5.35 ± 0.01	3.84×10^5^
Ti_45_Zr_20_Be_21_Co_14_	1875 ± 62	2101	6.4 ± 0.1	5.55 ± 0.01	3.38×10^5^
Ti_45_Zr_20_Be_19_Co_16_	1915 ± 45	1995	1.1 ± 0.1	5.62 ± 0.01	3.41×10^5^
Ti_45_Zr_18_Be_35_Co_2_	1885 ± 89	1855	0	4.78 ± 0.01	3.94×10^5^
Ti_45_Zr_16_Be_35_Co_4_	1952 ± 74	1952	0	4.83 ± 0.01	4.04×10^5^
Ti_45_Zr_14_Be_35_Co_6_	1988 ± 43	1988	0	4.99 ± 0.01	3.98×10^5^
(Ti_45_Zr_20_Be_35_)_98_Co_2_	1685 ± 94	1880	0.8 ± 0.1	4.77 ± 0.01	3.53×10^5^
(Ti_45_Zr_20_Be_35_)_97_Co_3_	1774 ± 32	1846	0.4 ± 0.1	4.96 ± 0.01	3.58×10^5^
(Ti_45_Zr_20_Be_35_)_96_Co_4_	1734 ± 49	1801	0.2 ± 0.1	5.00 ± 0.01	3.47×10^5^
(Ti_45_Zr_20_Be_35_)_94_Co_6_	1668 ± 68	1746	0.4 ± 0.1	5.01 ± 0.01	3.33×10^5^

**Table 5:** The values of *E*_g_ and *E*_x_ calculated by Kissinger and Moynihan equations.

Method	Ti_45_Zr_20_Be_35_		Ti_45_Zr_20_Be_31_Co_4_		Ti_45_Zr_16_Be_35_Co_4_		(Ti_45_Zr_20_Be_35_)_96_Co_4_	
	*E*_g_ (kJ/mol)	*E*_x_ (kJ/mol)	*E*_g_ (kJ/mol)	*E*_x_ (kJ/mol)	*E*_g_ (kJ/mol)	*E*_x_ (kJ/mol)	*E*_g_ (kJ/mol)	*E*_x_ (kJ/mol)
Kissinger	164	159	156	234	153	257	149	233
Moynihan	174	170	166	246	163	268	159	245

**Table 6:** The values of *E*_p1_ obtained using Kissinger, Moynihan, Ozawa, and Boswell methods.

Method	Ti_45_Zr_20_Be_35_	Ti_45_Zr_20_Be_31_Co_4_	Ti_45_Zr_16_Be_35_Co_4_	(Ti_45_Zr_20_Be_35_)_96_Co_4_
	*E*_p1_ (kJ/mol)	*E*_p1_ (kJ/mol)	*E*_p1_ (kJ/mol)	*E*_p1_ (kJ/mol)
Kissinger	169	201	191	193
Moynihan	180	213	202	204
Ozawa	172	203	193	194
Boswell	175	207	197	199

**Table 7:** The values of *n* derived from Figure 13.

*β* (K/min)	Ti_45_Zr_20_Be_35_	Ti_45_Zr_20_Be_31_Co_4_	Ti_45_Zr_16_Be_35_Co_4_	(Ti_45_Zr_20_Be_35_)_96_Co_4_
5	2.45	3.00	3.02	3.09
10	2.60	3.67	3.13	3.55
20	2.61	3.48	3.30	2.89
40	2.01	2.73	2.53	2.48

**Table 8:** The variation of corrosion potentials, pitting potentials, passive region widths, corrosion current densities, and polarization resistances of Ti_45_Zr_20_Be_35_, Ti_45_Zr_20_Be_31_Co_4_, Ti_45_Zr_16_Be_35_Co_4_, and (Ti_45_Zr_20_Be_35_)_96_Co_4_ BMGs.

Composition	*E*_corr_ (mV)	*E*_pit_ (mV)	*i*_corr_ (A/cm^2^)	*Ε*_pit_-*Ε*_corr_ (mV)	*R*_p_ (Ω)
Ti_45_Zr_20_Be_35_	−652	−122	8.5 × 10^−7^	530	4299
Ti_45_Zr_20_Be_31_Co_4_	−535	145	4.9 × 10^−7^	680	6556
Ti_45_Zr_16_Be_35_Co_4_	−551	103	5.9 × 10^−7^	654	5781
(Ti_45_Zr_20_Be_35_)_96_Co_4_	−501	74	8.5 × 10^−7^	575	3964

## Supplementary Materials

The following are available online at https://www.mdpi.com/1996-1944/13/1/223/s1, Figure S1: Illustration of the determination of critical diameter for glass formation.
