# Supplementary material for: Preparation, Characterization, and Properties of Novel Ti-Zr-Be-Co Bulk Metallic Glasses
Source: Materials (Basel). 2020 Jan 4;13(1):223. doi: 10.3390/ma13010223 (PMC6981410; doi:10.3390/ma13010223)
Supplement: Supplementary file 1 [file materials-13-00223-s001.pdf]

materials

Preparation, Characterization, and Properties of Novel Ti-Zr-Be-Co Bulk Metallic Glasses

Pan Gong [1,2], Fangwei Li [1] and Junsong Jin [1,*]

[1] State Key Laboratory of Materials Processing and Die & Mould Technology, Huazhong University of Science and Technology, No. 1037 Luoyu Road, Wuhan 430074, Hubei, China;
[2] State Key Laboratory of Advanced Design and Manufacturing for Vehicle Body, Hunan University, Changsha 410082, Hunan, China
* Correspondence: jsjin@hust.edu.cn; Tel.: +86-27-8754-3491

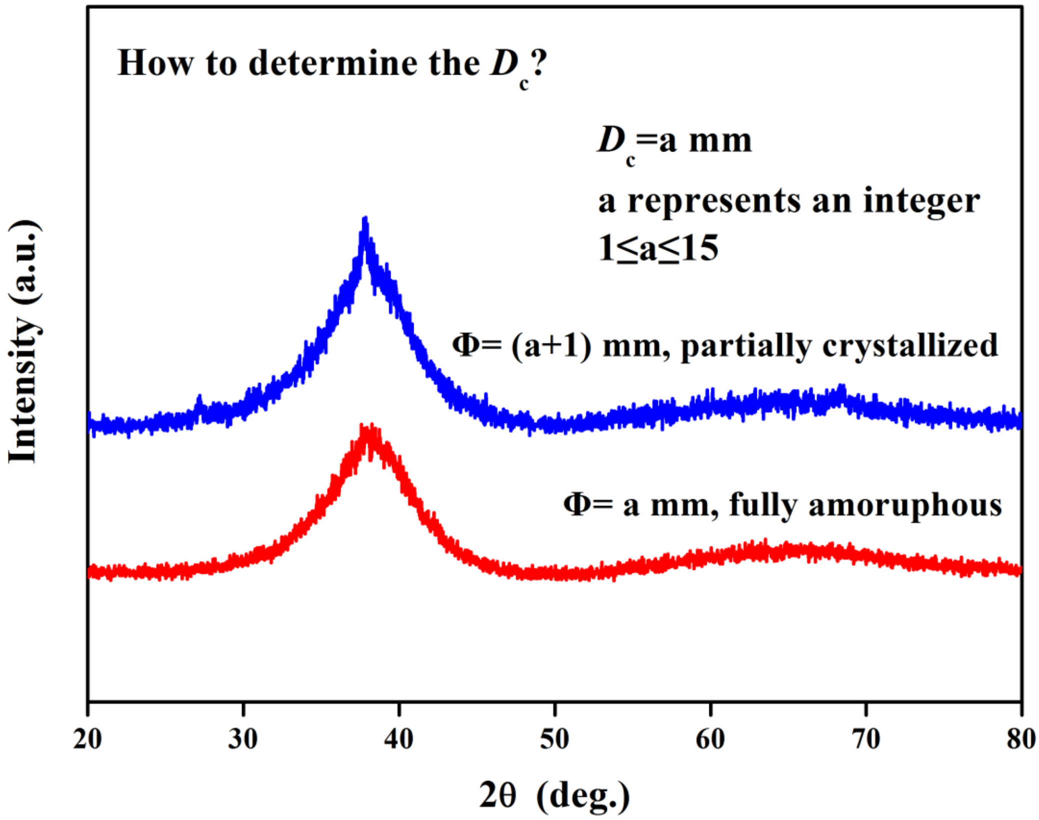

Figure S1. Illustration of the determination of critical diameter for glass formation.
